# Interesting statistics regarding the papers published in *Journal of Educational Evaluation for Health Professions* in 2017

**DOI:** 10.3352/jeehp.2017.14.36

**Published:** 2017-12-29

**Authors:** Yera Hur

**Affiliations:** Department of Medical Education, Konyang University College of Medicine, Daejeon, Korea; Hallym University, Korea

This year, from January 1 to December 27, a total of 112 papers were submitted to *Journal of Educational Evaluation for Health Professions (JEEHP)*. So far, 34 papers have been published this year, and 21 are in the processing stage. The acceptance rate is currently 27.4%, which is lower than the acceptance rate for 2016. Although this rate may change if more papers are accepted during the remaining days of 2017, I do not believe that the numbers will change drastically (Table 1). Raw data were available from Supplement 1.

Of the 34 papers that were published, there were 20 research papers, 4 opinion pieces, 3 papers each for educational/faculty development and reviews, 2 brief reports, and 1 editorial and technical report each. As shown in Fig. 1, the most frequently represented discipline was medical education (30.0%), followed by physical therapy (16.7%), and nursing (13.3%) and pharmacy (13.3%). I was happy to publications from a wide range of disciplines this year, and the portion of papers from each discipline did not significantly differ from the distribution last year. This may indicate that we are receiving papers from a great variety of disciplines.

It was interesting to see that 37.9% of the papers analyzed students. This may imply that researchers are focusing more on education from a student-centered perspective. However, simultaneously, this means that the rest of the papers analyzed a diverse variety of subjects, such as professors, clinical instructors or preceptors, and residents. With regard to the research content of the articles published this year (Fig. 2), 38.2% of studies included perception surveys, followed by papers reporting results from course evaluations (11.8%), assessment tools (11.8%), and licensing examination content development (8.8%).

Only 1 paper used qualitative method, while the remaining 96.4% employed quantitative analysis. Although *JEEHP* focuses on the evaluation of education, it would be nice to see more papers using qualitative methods or a mixture of qualitative and quantitative methods.

I examined the number of authors from various countries in papers published this year in *JEEHP*. Korean authors accounted for the largest portion of papers (35.3%), followed by authors from the United States (20.6%), Australia (8.7%), Spain (8.8%), Iran (5.9%), and Japan (5.9%), with 1 paper each written by authors from China, France, India, Nepal, and Palestine (Fig. 3).

The number of authors ranged from 1 to 10. The most frequent number of authors was 3 (6 articles, 17.6%), followed by 5 and 6 authors (14.7%). Only three papers were written by a single author (8.8%), implying that the articles mostly reflected collaborative research (Fig. 4).

Additionally, articles were analyzed according to both the number of authors and the number of countries of those authors. Most articles were written by multiple authors from the same country (Fig. 5). Six articles were written by 3 authors from 1 country, and 1 article was written by 10 authors from the same country. Only 3 papers had authors from multiple countries (8.8%).

*JEEHP* provides a link to see which articles were the most viewed quarterly. The most frequently accessed article was “Reliability of a viva assessment of clinical reasoning in an Australian pre-professional osteopathy program assessed using generalizability theory” by Vaughan et al. [1], which was published in January this year (accessed 11,913 times). The next most-viewed article was “Effect of practical training on the learning motivation profile of Japanese pharmacy students using structural equation modeling” by Yamamura and Takehira [2], which was published this February (accessed 11,339 times). This article was also the most downloaded article in 2017 (downloaded 223 times), and has already been cited by another article. This is a remarkable number of accesses because the papers were published just this year. In fact, the top 10 most frequently accessed articles were all published in 2017. I believe that *JEEHP* being indexed in the Emerging Sources Citation Index (December 2015), becoming a Medline journal, and being visible in the search results in SCOPUS (March 2016) have been very helpful in this regard. The article “Utility of eye-tracking technology for preparing medical students in Spain for the summative objective structured clinical examination” by Sanchez-Ferrer et al. [3], which was published just last month, has already been downloaded 65 times.

To summarize, I was happy to see that a wide variety of disciplines was represented in papers in *JEEHP* from various regions of the world and that the articles have been widely accessed. Nonetheless, the journal will be more helpful to its readers if: (1) more studies use qualitative methods or a mixture of quantitative and qualitative methods of assessment, (2) more studies reflect cooperative research done by authors from multiple countries, and (3) more original research articles are published. Most of all, it is not easy to maintain the low acceptance rate and high quality of the journal, but doing so is essential for the journal over the long term. I hope to see *JEEHP* indexed as a Science Citation Index journal in the very near future.

## Figures and Tables

**Fig. 1.**
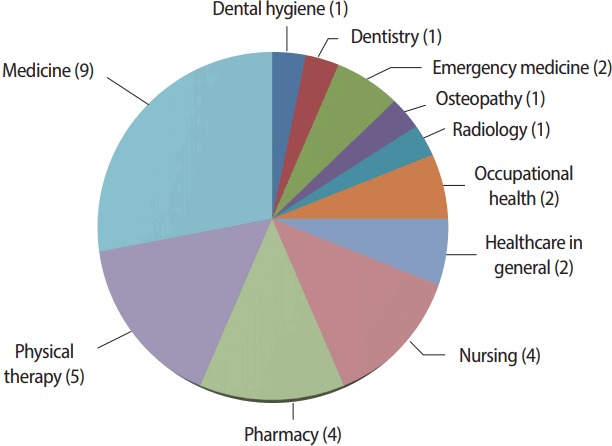
Number of articles published in 2017 by discipline.

**Fig. 2.**
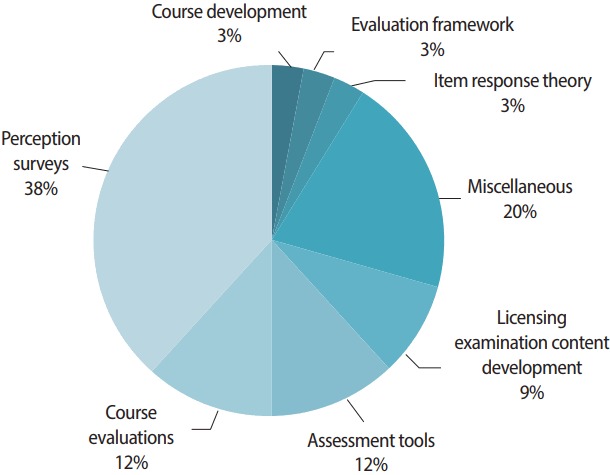
Number of articles published in 2017 by research content.

**Fig. 3.**
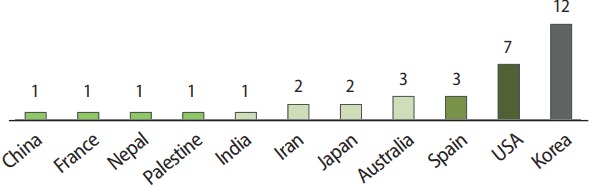
Number of articles published in 2017 by the country of the first author.

**Fig. 4.**
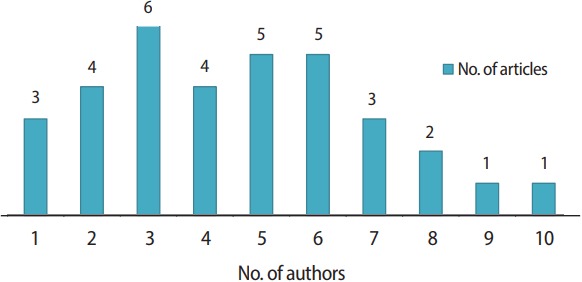
Number of authors of articles published in 2017.

**Fig. 5.**
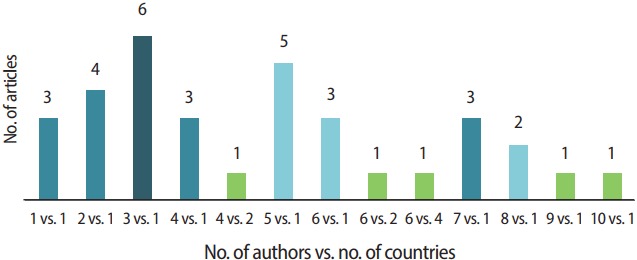
Number of articles published in 2017 by the number of authors versus number of countries.

**Table 1.** Article acceptance rate in 2016 and 2017

	Year	
	2016	2017
Articles accepted	39	34
Articles withdrawn	0	2
Articles rejected	90	88
Acceptance rate (%)	30.2	27.4

## Supplementary material

Supplement 1. Data files are available from https://doi.org/10.7910/DVN/S9FG5U
